# Rapid Rapamycin-Only Induced Osteogenic Differentiation of Blood-Derived Stem Cells and Their Adhesion to Natural and Artificial Scaffolds

**DOI:** 10.1155/2017/2976541

**Published:** 2017-07-26

**Authors:** Carpentieri Arianna, Cozzoli Eliana, Acri Flavio, Ranalli Marco, Diedenhofen Giacomo, Scimeca Manuel, Bonanno Elena, Gambacurta Alessandra

**Affiliations:** ^1^Biochemistry Laboratory, Department of Experimental Medicine and Surgery, University of Rome “Tor Vergata”, 00133 Rome, Italy; ^2^Baxter Healthcare Ltd, Caxton Way, Thetford, UK; ^3^Department of Biomedicine and Prevention, University of Rome “Tor Vergata”, 00133 Rome, Italy; ^4^OrchideaLab S.r.l., Via del Grecale 6, Morlupo, Rome, Italy; ^5^NAST Centre for Nanoscience, University of Rome “Tor Vergata”, 00133 Rome, Italy

## Abstract

Stem cells are a centerpiece of regenerative medicine research, and the recent development of adult stem cell-based therapy systems has vigorously expanded the scope and depth of this scientific field. The regeneration of damaged and/or degraded bone tissue in orthopedic, dental, or maxillofacial surgery is one of the main areas where stem cells and their regenerative potential could be used successfully, requiring tissue engineering solutions incorporating an ideal stem cell type paired with the correct mechanical support. Our contribution to this ongoing research provides a new model of in vitro osteogenic differentiation using blood-derived stem cells (BDSCs) and rapamycin, visibly expressing typical osteogenic markers within ten days of treatment. In depth imaging studies allowed us to observe the adhesion, proliferation, and differentiation of BDSCs to both titanium and bone scaffolds. We demonstrate that BDSCs can differentiate towards the osteogenic lineage rapidly, while readily adhering to the scaffolds we exposed them to. Our results show that our model can be a valid tool to study the molecular mechanisms of osteogenesis while tailoring tissue engineering solutions to these new insights.

## 1. Introduction

Regenerative medicine is an important and relevant scientific research field whose focus on developing cell-based therapeutic approaches to regenerate damaged tissues, or even replace whole organs, has attracted scientists from around the world [1]. One of the main areas where stem cells and their regenerative potential could be used successfully is orthopedic, dental, and maxillofacial surgery [2, 3]. Problems such as the treatment of fractures involving extensive bone damage or a particularly sensitive localization are a complex challenge for surgeons, and there are some pathological conditions that are treatable only insufficiently with conventional implants and surgical procedures [4–6].

The solution can be found in tissue engineering, where cells are combined with a three-dimensional matrix to compose a tissue-like construct to substitute lost tissues, or even whole organs [1]. Adult stem cells have shown remarkable plasticity, and they have been extensively studied for their potential applications in tissue engineering [6, 7].

Today, there are many sources from which it is possible to draw adult stem cells, such as bone marrow, adipose tissue, periosteum, human umbilical cord, and mesenchymal tissue [8–11].

Presently, mesenchymal stem cells seem to be the most promising candidates for bone regeneration due to their excellent osteogenic differentiation capacity, but they are not without drawbacks as these cells are relatively difficult to expand in vitro, and they lose their regenerative potential during in vitro passages [12, 13]. Another factor that enormously restricts the use of stem cells as a routine therapeutic tool is that we have little knowledge about the specific pathways they use during differentiation, bone healing, and revascularization [6].

A further prominent aspect in the study of osteogenic differentiation from stem cells is the importance of physical and/or mechanical supports. The role of the scaffold is to support cell growth and differentiation and to control interactions of cells within the biological milieu, while maintaining the mechanical and physical properties required for the selected application [14]. Although implants are successful, it takes about 4–6 months for the healing and integration of implants within the existing bone to occur [14]. Thus, the real challenge in bone regeneration and repair lies in accelerating healing and reducing the recuperation time, since the lack of cell adhesion to substrates commonly used in orthopedic and dental implants remains the leading cause of slower integration and subsequent recovery time [3, 14].

The ideal combination of both the regenerative properties of stem cells and the mechanical support provided by implants and scaffolds would require a simple stem cell production method paired with good adherence to the scaffold [15]. Any simplification of the production process makes the eventual procedure more time- and cost-effective, while good adhesion ensures a greater implant success rate. Finding the right combination between stem cell type and three-dimensional support/scaffold remains one of the main challenges of regenerative medicine and is particularly relevant to the field of bone tissue regeneration [16, 17]. This “winning couple” should be suited for both therapeutic and research purposes, in order to elucidate the mechanisms involved in tissue regeneration while providing new tools to the clinician.

However, it is clear that much more research has to be done before we can reach such an ideal stage, as regenerative therapies for bone reconstitution require a deeper understanding of both the biological and the mechanical conditions involved. As with all research endeavors, the creation of new models that may facilitate these studies remains a top priority.

Recently, rapamycin, a selective mTOR (mammalian target of rapamycin) inhibitor, has been shown to induce osteoblastic differentiation in human embryonic stem cells [18, 19]. This molecule has been shown to act on the PI3K/AKT/mTOR signaling pathways, inducing the upregulation of the early osteogenic markers BMP 2 and Runx2 and modulating the BMP/Smad signaling in human embryonic stem cell (hESC), as well as being important in the regulation of self-renewal and differentiation [20, 21]. Rapamycin is already approved by the FDA for use as a therapeutic agent for humans, making it potentially translatable to orthopedic applications. In fact, it has already shown promise of future applicability in bone regeneration both in vitro and in vivo [22]. Taking this into consideration, we were thus intrigued by the possibility of further applications of this specific molecule within the setting of a new “in vitro” model of bone differentiation. Having already demonstrated, the plasticity of blood derived-stem cells (BDSCs) [23–26] and knowing their potential applications in bone tissue repair, we tried to develop a reproducible and defined method to investigate their potential for osteogenic/cellular differentiation.

In this paper, we propose an osteogenic differentiation model that makes use of both rapamycin as a single inductor and bone matrix scaffolds. The results obtained show that not only the timing of commitment towards the osteogenic lineage is shortened compared to the literature [27, 28] but also it is possible to follow this process through a simple confocal microscopy analysis.

Furthermore, we have also shown the ability of BDSCs to adapt and adhere to different types of scaffolds approved for medical use, particularly in orthopedic and dental surgery.

## 2. Materials and Methods

### 2.1. Stem Cells Preparation

Human BDSCs were obtained by blood samples as previously described [23–26]. Briefly, the nucleated blood cell fraction was isolated by ammonium chloride incubation (dilution 1 : 3 in NH_4_Cl 1 M), centrifuged at 400*g*, and washed several times with phosphate-buffered saline (PBS), pH 7.2 (Oxoid, Hampshire, England, number BR0014G) to remove the majority of erythrocytes. Cells were then resuspended in 5 ml PBS and incubated for 72 h at 37°C in the presence of 50 nM macrophage colony-stimulating factor (Sigma-Aldrich, St. Louis, MO, number M9170), and 5 *μ*M gentamicin sulphate (BioWest, Nuaillé, France, number L0012).

### 2.2. Osteogenic Differentiation of BDSCs

To promote osteogenic differentiation in vitro, the human BDSCs were plated on a collagenated 24-multiwell plate. The plate coating protocol required a 0.1 mg/ml type I rat tail collagen, high concentration solution (BD Pharmigen, USA, cat. number 354249), added to the 24-well plates, and incubated for 1 h at 37°C. After incubation, the plates were washed three times with PBS and kept at +4°C until use.

We used different procedures to differentiate BDSCs:

- Commercial kit: The cells were differentiated using the “Human Mesenchymal Stem Cell Functional Identification Kit” (R&D systems, number SC006) according to the manufacturer's protocol.
- Rapamycin: The cells were grown in a 24-well plate for 10 days in a differentiation medium composed by DMEM-F12 supplemented with 15 mM HEPES and 2 mM L-glutamine (Lonza, Swiss, cat. number BE12-719F), 10% FBS (Lonza, Belgium, cat. number DE14-830F), 1% penicillin (100 units/ml)/streptomycin (100 mg/ml) (Lonza, Belgium, cat. number DE17-602E), 1% nonessential amino acids (Biowest, cat. number X0557–100), and Rapamycin, as osteogenic inductor, at a final concentration of 10 nM (Sigma-Aldrich, cat. number R0395).

#### 2.2.1. Rapamycin and Scaffolds

The cells were grown for 10 days in the presence of several types of scaffolds in the same differentiation medium as described above.

### 2.3. Scaffold Types

#### 2.3.1. Bone Scaffold

We used the BIO-OSS a spongious bone substitute of bovine origin produced by Geistlich Biomaterials. The granule size was 0.25 mm–1 mm/0.5 g [29].

#### 2.3.2. Titanium Scaffold

We used two titanium commercial products. The first (type 1) from CAMLOG, Alta-Tech Biotechnologies Srl. was obtained by classical endosseous implants and cut into thin slices (suitable for the 24-well plates) with extrathin cutting discs nonpolluting inline © (B.M. DENTALE S.a.s.). The second (type 2) was derived from a titanium grill used in the dental field for guided bone regeneration (TITANGUIDE), produced by Prodent Italia S.r.l. In this case, the original surface of each plate (20.5 mm × 30 mm × 0.13 mm) possesses numerous microholes (Ø 0.7 mm) and was also cut to obtain rectangles (5 × 4 mm average dimensions) to perform the experiments in the 24-well plates.

Titanium scaffold treatment protocol, to avoid particulates, autoclave sterilization followed by an ultrasonic bath for 5 min in deionized H_2_O, and then for 5 min in H_2_O_2_ at 120 *v*/*v*, and again for 10 min in deionized H_2_O, was performed, as decribed by Di Silvestro et al. [30]. Furthermore, to avoid alterations of the superficial layer of the scaffolds, an important factor in cell/surface interaction, particular care was taken in performing any and all manipulations of the titanium scaffolds using only plastic or pure titanium instruments.

### 2.4. Alizarin Red S Staining

The presence of calcium phosphate deposits in BDSCs following differentiation was evaluated by staining with Alizarin Red S (Sigma-Aldrich, Germany, cat. number A5533-25G). The cells were fixed with 4% [*v*/*v*] formaldehyde, stained with 1% [*v*/*v*] Alizarin Red S, and incubated at room temperature in the dark for 20 minutes. The excess of dye was removed with distilled water, and the cells were observed by optical microscopy.

### 2.5. Immunofluorescence Analyses

The cells were washed twice with PBS and fixed on a glass slide with 4% formalin (ScyTek Laboratories, Logan, UT) for 30 min at room temperature (RT), then washed with PBS, and permeabilized/blocked with 0.3%Triton X-100, goat serum 5%, and 1% BSA in PBS for 45 min at RT. After adding blocking solution, the washed cells were incubated with the primary antibody mouse anti-osteocalcin (1 : 100 R&D System 962643) overnight at 4°C. Subsequently, the cells were incubated for 1 hour in the dark at RT with the secondary antibody anti-mouse Alexafluor 488 (1 : 1000) (Invitrogen, Life Technologies Corporation, NY) and with 1 *μ*g/ml of 40,6-diamidino-2-phenylindole (DAPI, Sigma) to stain cells nuclei.

### 2.6. Time Course Experiments on Titanium

We performed a time-dependent analysis to evaluate cellular growth and adhesion on both types of titanium. Briefly, seven type 2 titanium scaffolds were placed in seven separate wells, all treated as previously described. Every day for a week, one scaffold was taken and fixed in formalin 4%, washed with PBS after 20 minutes, and prepared for confocal microscopy analysis in order to have a full picture of the cellular growth.

Two types of analyses were performed:

- Densitometric analysis: To determine the cells' adhesion to the titanium scaffold, we calculated the increase in average luminosity of DAPI over time executed on the blue fluorescence channel using the Nikon EZ-C1 Viewer software.

To verify that data obtained from the analysis was not simply influenced by an increase of background noise with a correlated increase of average luminosity, we controlled the intensity of luminosity of the same channel along a random direction of the image.

(ii) Coverage analysis: To calculate the percentage area of scaffold covered by cells, an analysis of intensity of luminosity per single pixel was performed using the NIS Elements Viewer provided by Nikon (Copyright © 2005 Macrovision Corporation). Based on a control sample, the lowest and highest luminosity values were ignored in order to eliminate both, the background noise, and the peaks with a too high luminosity levels.

All images have been shot at 12 bit, therefore presenting a resolution of 4096 (2^12^) luminosity levels per pixel. On a scale from 0 to 4095, all pixels with an ADC (analog to digital converter) resolution below 156 units and all those with an ADC above 2000 were ignored. All signals within this window have been recorded, yielding a percentage of scaffold surface area covered by cellular growth.

### 2.7. Protein Extraction and Western Blot Analysis

Cells were homogenized directly into the following buffer: Tris 50 mM, NaCl 150 mM, EDTA 10 mM, and Triton-X 1% and centrifuged at 10,000*g* for 10 min. Protein concentrations were determined by the Bradford assay. Proteins were resolved by 16% SDS-PAGE, electrotransferred on PVDF membranes (Amersham™ Hybond™, GE Healthcare Life Science, cat. number 28906837), and blocked with 5% [*v*/*v*] nonfat dry milk/0.1% [*v*/*v*] TBS-T. The blots were probed with the following primary antibodies: mouse monoclonal anti-osteocalcin (1 : 500) (abcam-ab13420) in 5% BSA/TBS-T 0.1% and mouse monoclonal anti-beta actin (1 : 10,000) (sigma-aldrich-A5541).

Membranes were then incubated with the appropriate horseradish peroxidase-conjugated donkey anti-mouse secondary antibody (1 : 5000; Jackson), and the reaction was detected with the Western lighting Plus ECL (Perkin Elmer, Waltham, MA, USA).

### 2.8. Scanning Electron Microscopy (SEM) and Energy Dispersive X-Ray (EDX) Microanalysis

Small scaffold supports were fixed in 4% (*v*/*v*) paraformaldehyde and postfixed in 2% osmium tetroxide. After washing with 0.1 M phosphate buffer, the sample was dehydrated by a series of incubations in 30%, 50%, and 70% (*v*/*v*) ethanol. Dehydration was continued by incubation steps in 95% (*v*/*v*) ethanol, absolute ethanol, and acetone. Critical-point drying (Agar Scientific, ElektronTechnology UK Ltd) with supercritical CO_2_ was then performed to prevent cell deformation. Surfaces of the scaffolds were coated with gold and scanned using SEM LEO 1450VP (Carl Zeiss Meditec, Germany) [31]. EDX microanalysis was performed on exosome using a liquid N_2_-cooled Si detector with a super-ultrathin Be window. Spectra were collected by a SEM LEO 1450VP scanning electron microscope at acceleration voltage of 5 KeV employing an area scan mode (640 × 640 *μ*m sampling area), 300 s acquisition time, and 32–37% detector dead time. Analysis of acquired spectra was performed under a nonstandard mode using atomic number-absorption-florescence correction (ZAF) methods using Inca Energy software (Oxford Instruments Ltd., High Wycombe, UK; Si(Li) detector, ATW—atmospheric thin window, resolution 133 eV for MnK*α* at 10000 counts).

### 2.9. Transmission Electron Microscopy (TEM) and EDX Microanalysis

Cells were fixed in 4% paraformaldehyde, postfixed in 2% osmium tetroxide, and embedded in EPON resin for morphological studies. After washing with 0.1 M phosphate buffer, the sample was dehydrated by a series of incubations in 30%, 50%, and 70% ethanol [32]. Dehydration was continued by incubation steps in 95% ethanol, absolute ethanol, and hydropropyl methacrylate, and then samples were embedded in Epon (Agar Scientific, Stansted Essex CM24 8GF, United Kingdom). After incubation, cells were cut and stained with heavy metal solutions as described by Reynolds [33]. For the EDX microanalysis, 100 nm thick unstained ultrathin sections were placed on specific copper grids. The EDX spectra were acquired on matrix vesicles by a Hitachi 7100FA transmission electron microscope and an EDX detector (Thermo Scientific, Waltham, MA, USA) at an acceleration voltage of 75 KeV. Spectra were semiquantitatively analyzed by the Noram System Six software (Thermo Scientific, Waltham, MA, USA) using the standardless Cliff-Lorimer k-factor method [34]. The EDX microanalysis system was calibrated using the X-ray microanalysis standard (Micro-Analysis Consultants Ltd, Cambridgeshire, UK).

## 3. Results and Discussion

### 3.1. BDSCs Successfully Differentiate into Osteogenic Tissue

**Figure 1:**
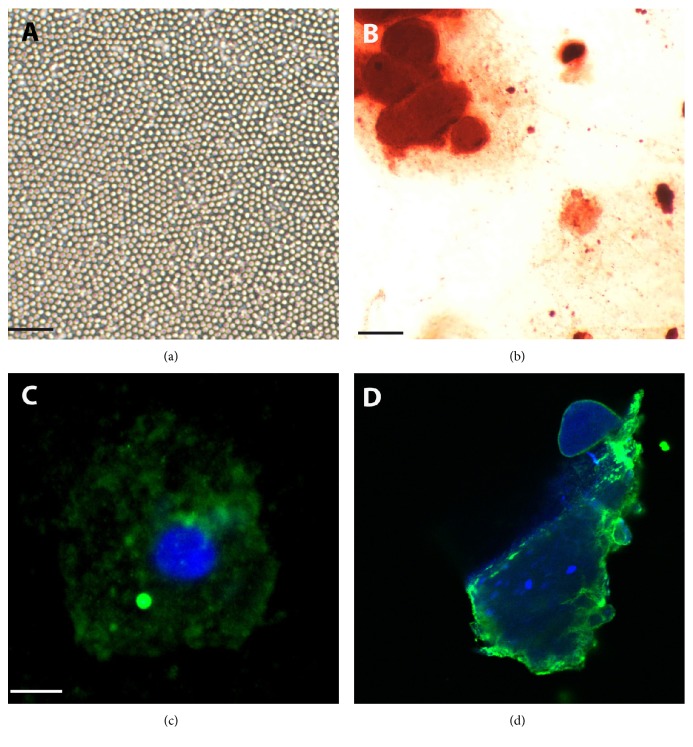
Blood derived stem cells' (BDSCs) osteogenic differentiation with rapamycin. (a) BDSCs before starting the differentiation protocol. The cells show typical morphological features of stemness such as small size, roundish shape, and, in vitro, a disposition to a “string of pearls” appearance. (b) Alizarin Red S staining on BDSCs after 10 days of osteogenic differentiation, to evaluate inorganic calcium phosphate deposition. (c) Visualization by immunofluorescence analysis through confocal microscopy of a single differentiated cell (60x). (d) Image from three-dimensional stack analysis by confocal microscopy of blood-derived stem cells after ten days of osteogenic differentiation (in green, osteocalcin; in blue, DAPI: merged; (a), (b) 100 *μ*m, (c) bar = 8 *μ*m).

The BDSCs derived from 72 h of deprogramming and expansion in PBS, supplemented with MCSF and gentamicin sulphate [23–26], were plated and presented typical morphological features of stemness such as reduced small size, roundish shape, and, in vitro, a disposition to a “string of pearls” morphology (Figure 1(a)). Once we confirmed the achievement of these morphological changes, our BDSCs were ready to demonstrate their potential for osteogenic differentiation.

We first tested this by utilizing the commercial kit already used in the majority of works on stem cell bone differentiation as our inducer. Our BDSCs were responsive to this treatment and acquired osteoblast-like features within 15 days (data not shown). The commercial differentiation kits are mainly composed by a cocktail of dexamethasone, ascorbic acid, *β*-glycerophosphate, and various added excipients. The mechanisms underlying the kit-mediated osteogenic differentiation are only partially known and understood, while the combination of substances used is not approved for human therapy yet. For these reasons, we believe that new differentiation protocols are necessary to put more circumscribed molecular mechanisms into play, with the aim of achieving a higher translational potential to in vivo application.

### 3.2. Rapamycin Alone Allows for Efficient and Rapid Differentiation

We then attempted this differentiation anew, using only rapamycin 10 nM as an inducer [20–22]. After ten days of rapamycin treatment, we stained the cells with Alizarin Red S to detect the presence of inorganic calcium phosphate deposits and to confirm that osteogenic differentiation occurred (Figure 1(b)). Although the detection of mineralization in confluent monolayers by Alizarin Red S is often considered sufficient evidence to demonstrate a completed osteogenic differentiation [35], we also performed an immunocytochemical, immunofluorescence analysis for detecting osteocalcin, a typical osteogenic marker, as further evidence of the successfully completed process (Figures 1(c) and 1(d) and SD1 available online at https://doi.org/10.1155/2017/2976541). This data suggests that our cells can differentiate within 10 days under the exclusive guide of Rapamycin, making it a good alternative to the commercial kit.

### 3.3. Bone Scaffolds Improve Rapamycin Protocol Yield

**Figure 2:**
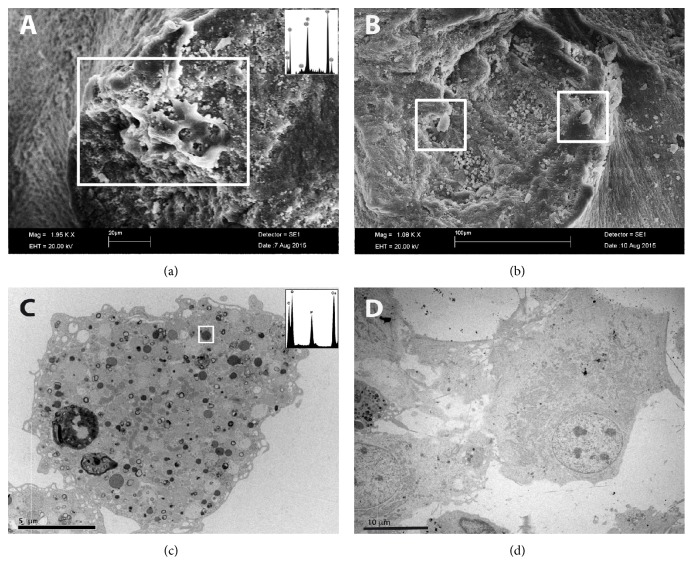
Electron microscopy demonstrating scaffold colonization of BDSCs differentiated with rapamycin, on bone scaffold. (a) Higher magnification of area indicated in the rectangle displays cells forming tissue-like agglomerates. Notably, electron micrograph captures an osteoblast matrix vesicle just as it initiated the exocytosis of hydroxyapatite (EDX spectrum). (b) Rectangles indicate differentiated osteoblast-like cells as shown by transmission electron microscopy analysis. (c) Higher magnification shows electron-dense granules within the mitochondria and the presence of calcium and phosphorus calcium phosphate aggregates typical of an osteoblast (EDX spectrum). (d) TEM image shows a typical osteoblast cell with flat lamellipodia and dendritic filopodia (bar = 20 *μ*m in (a), 100 *μ*m in (b), bar = 5 *μ*m in (c), and 10 *μ*m in (d)).

Once we demonstrated, the ability of BDSCs to differentiate into osteoblast-like cells using rapamycin, we performed the same differentiation experiments in the presence of deproteinized and decellularized bovine bone matrix (SD 2). Our aim was to verify whether these cells were able to undergo osteogenic differentiation when in contact with a scaffold and whether the presence of the matrices improved the process. After the full ten-day treatment, we performed an ultrastructural analysis by SEM and TEM (Figures 2(a) and 2(b) and Figures 2(c) and 2(d), resp.), cytochemistry, and Western blot analysis for osteocalcin (SD 2) to verify our hypothesis.

SEM evaluation confirmed that the BDSCs cultured in the presence of rapamycin and bone matrix scaffold underwent morphological changes into osteoblast-like phenotypes, as shown in Figure 2(a), showing extended osteocalcin deposits. In addition, we captured the calcium phosphate-containing exosomes on the surface of osteoblast-like cells (riferimento imagine SEM con lo spettro EDX).

TEM analysis also showed that osteoinduced BDSCs had a bulging cell body abundant in cytoplasm showing low N/P ratio. The cells extended many flat lamellipodia and dendritic filopodia with structural surface modifications, presenting many microvilli (Figures 2(c) and 2(d)).

The cytoplasm was rich in ribosomes and membranous organelles such as a well-developed ER and Golgi apparatus and many mitochondria exhibiting the orthodox configuration, with the inner compartment filled with abundant electron-dense matrix. Furthermore, EDX microanalysis allowed us to demonstrate the presence of calcium phosphate-containing vesicles and calcium-containing granules within mitochondria. These “matrix vesicles” have been implicated in the mineralization of cartilage, bone, and dentin [14, 15, 36]. The observation that calcium phosphate and/or calcium is present both within mitochondria and intracellular vesicles suggests that a mechanism may exist in osteogenic cells by which ionic calcium (and perhaps phosphate) are transferred from mitochondria to intracellular vesicles, possibly via a simple process such as diffusion. This mechanism may play a role in bone apatite formation [37].

The aim of this new experimental protocol was to use a single molecule as an inducer and provide a simpler model to investigate the mechanisms underlying the osteogenesis of stem cells. The addition of bone scaffolds represented an improvement, resulting in a more uniform osteogenesis and exploring the ability of our cells to attach to and build on a bone matrix structure. These same bone scaffolds are routinely used in orthodontic procedures for numerous applications [38].

### 3.4. Rapamycin Protocol Also Effective on Titanium Scaffold

Once the ability of BDSCs to undergo osteogenic differentiation was confirmed, and a simplified differentiation protocol using rapamycin and bone matrix was established, a further step in our investigation was done in order to apply our protocol to a completely different material for osteogenic differentiation, namely, titanium.

**Figure 3:**
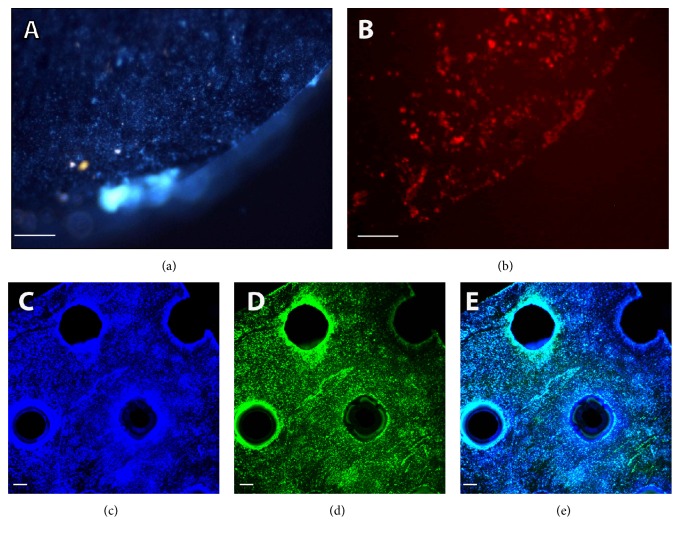
BDSCs osteogenic differentiation with rapamycin and titanium scaffolds. Nuclear and Alizarin Red S stainings on type 1 titanium after differentiation by confocal microscopy analysis ((a) and (b), resp.). Nuclear staining, osteocalcin expression, and merge analysis on type 2 titanium scaffold ((c), (d), and (e), resp.) (osteocalcin green, DAPI blue; (a), (b) bar = 10 mm; (c), (d) 200 *μ*m 4x).

**Figure 4:**
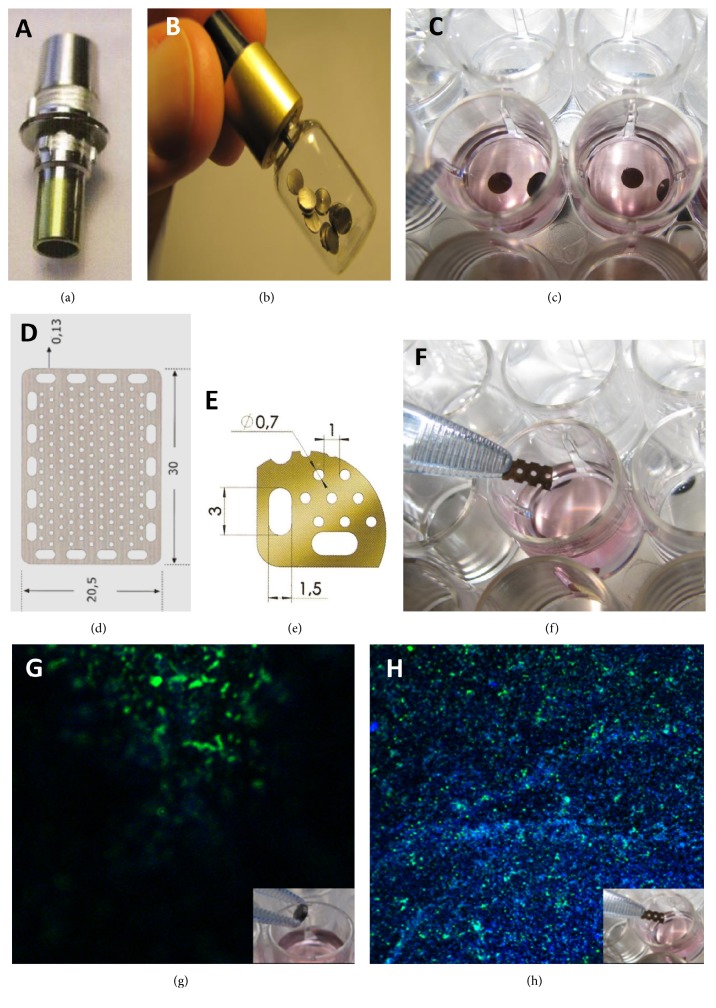
Titanium scaffolds: Titanium type 1. (a) The original endosseous implants before cutting, (b) titanium after cutting and sterilization, (c) titanium type 1 in a 24-well plate before osteogenic differentiation experiments. Titanium type 2. (d) The original titanium grill before cutting. (e) The dimensions of the different perforations. (f) Titanium type 2 in a 24-well plate before osteogenic differentiation experiments. (g) Titanium type 1 immunofluorescence images by confocal microscopy, the low resolution of the osteocalcin and nuclei signals are evident (green and blue, resp.). (h) Titanium type 2 immunofluorescence images by confocal microscopy, it is evident how the more flat surface of this scaffold is better suited for confocal microscopy analysis.

Two titanium scaffolds were taken into consideration for these further experiments: the first being slices sampled from a titanium cylinder (type 1) and the second being pieces of a perforated sheet (type 2) both already commercially available as implants for orthodontic application in humans. After repeating our analysis with Alizarin Red S staining and immunocytochemical analysis for detecting osteocalcin, we found that cells successfully adhered to both. However, type 2 was selected as the more ideal scaffold as it allowed for better imaging, since the surface of the type 1 titanium slices had proven to be more irregular than expected and their display by confocal microscopy showed unsharp images due to cells being located on different planes (Figures 3 and 4). Time course experiments for osteocalcin expression were then performed to better evaluate the progression of colonization during the differentiation process.

**Figure 5:**
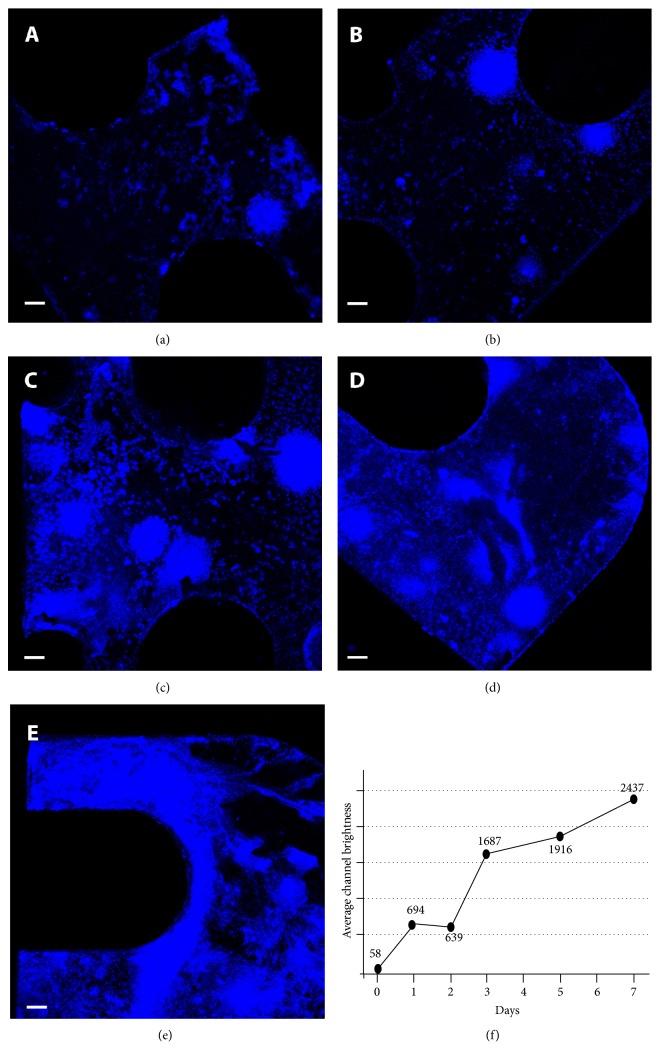
Time course experiments to detect the BDSCs capacity to adhere to the titanium scaffold. The increasing blue signal (DAPI) along time course analysis shows the cells' adhesion and growth on titanium ((a) = after 1 day, (b) = 2 days, (c) = 3 days, (d) = 5 days, (e) = 7 days). (f) shows a graphical representation of (a)–(e) time course analysis performed by Nikon EZ-C1 viewer software.

**Figure 6:**
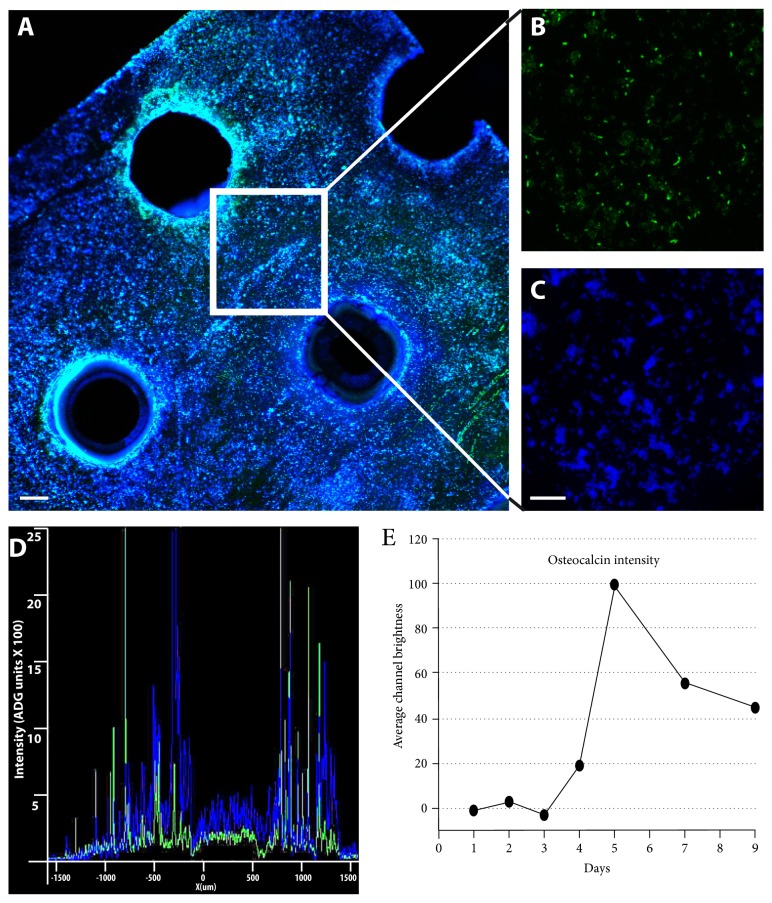
Analysis to detect BDSC capacity to differentiate on titanium scaffolds. (A) Merge analysis on type 2 titanium scaffold by osteocalcin expression ((B) osteocalcin green) and nuclear staining ((C) DAPI blue) to demonstrate BDSCs osteogenic differentiation. (D) shows a graphical representation of the two channel light intensity signals (expressed in ADG units ×100). It is evident how the blue peaks (nuclear signal) overlap the green peaks (osteocalcin signal). (E) shows time course experiments for osteocalcin expression (expressed as average channel brightness) ((A): bar = 200 *μ*m, (B), (C): bar = 50 *μ*m).

To estimate the area of scaffold material coated by the cells, we set up a double analysis, combining densitometry with overlapping fluorescence signals as measured by two software that allowed to calculate the percentage of area covered by the cells during their growth. This analysis was optimized by evaluating the intensity of image pixel brightness, removing the lowest light intensities due to round noise, and the highest due to artifacts (SD 3). Making a time-dependent analysis of the colonization process using two different confocal microscopy software allowed us to compare the percentage of metal surface covered by cells with the cell proliferation rate (Figure 5). The results show that not only BDSCs were able to colonize up to 90% of the titanium scaffold during their osteogenic reprogramming but also that the maximum peak of differentiation is obtained already during the first week. Furthermore, this simple imaging protocol allowed us to follow the differentiation process over time. The results obtained by overlaying the two signals in our experiments confirm the presence of the nuclei (in blue) and highlight the secreted osteocalcin (in green), enabling us to pinpoint how peak osteocalcin expression is reached as early as five days postinduction (Figure 6). In both cases, the differentiation timeframe of our experiments is significantly shorter than all those hitherto reported in similar works [39].

It is well known that the surface properties of a scaffold material can regulate stem cell fate [40–43] and, for example, induce osteoblast differentiation [44, 45]. Theoretically, an ideal titanium implant would favor stem cell differentiation into mature osteoblasts for direct bone apposition. However, the development of such an ideal implant would require a more extensive investigation of the processes of attachment, colonization, and differentiation of these stem cells. We believe that our model may provide a first step in that direction.

## 4. Conclusion

With this work, we demonstrated that BDSCs are able to differentiate toward the osteogenic lineage not only by using a standard commercial kit but also by using rapamycin as single inducer molecule, both by itself and in the presence of bone or titanium scaffolds. We have shown that these cells are able to attach, proliferate, and differentiate on a nonpremodified, smooth titanium plate and that this scaffold does not interfere with the differentiation process. All of the materials used in these new protocols have been selected with an eye towards applicability in human therapy. This simple procedure, paired with its rapid differentiation time, might prove to be a valid model to study the molecular mechanisms of osteogenesis while tailoring tissue engineering solutions to these new insights. BDSCs could prove to be a valid addition to the regenerative medicine toolbox, with the ultimate goal of creating new treatments to stimulate bone regeneration and improve prosthetic implantation.

## Supplementary Material

The information of supplementary materials are as follows: Supplemental Data Figure 1. Three-dimensional stack by Confocal microscope of Blood Derived Stem Cells after ten days of osteogenic differentiation with rapamycin. In green osteocalcin. In blue the nuclear marker DAPI. Supplemental Data Figure 2. Osteogenic differentiation of Blood Derived Stem Cells by rapamycin on bone scaffolds. Panel A: BDSCs during differentiation (bar = 100 um). Panel B: Alizarin Red staining on BDSCs after ten days of differentiation (bar = 100 um). Panel C: Western blot analysis for osteocalcin (12 kDa) and b-actin (42kDa) as control on BDSCs and differentiate cells respectively. Supplemental Data Figure 3. Analysis on titanium of the covered area by the Blood Derived Stem Cells. Panel A: Analysis with software NIS Elements Viewer to calculate the percentage of titanium area covered by BDSCs (four time points are represented at 24h, 72h, 5 days and 7 days of osteogenic induction). Panel B: Histograms in percentage of increase over time of titanium coverage area.
